# Early versus late tracheostomy: what do patients want?

**DOI:** 10.1186/s13054-023-04443-4

**Published:** 2023-04-19

**Authors:** Anna-Liisa Sutt, John F. Fraser

**Affiliations:** 1 Critical Care Research Group, Adult Intensive Care Services, The Prince Charles Hospital, Rode Road, Chermside, Brisbane, QLD 4032 Australia; 2 Faculty of Medicine, University of Queensland, St Lucia, Brisbane, QLD 4072 Australia

The debate around the best timing for performing a tracheostomy remains active. The recent meta-analysis by Premraj et al. [1] failed to find an association between timing of tracheostomy and mortality, ICU or hospital length of stay (LOS), or neurological outcomes (mRS) when analysing data from > 17,000 patients. The main outcomes of interest in this topic have remained the same for decades. Back in 1993 Heffner [2] found it surprising no clear consensus existed on timing of replacing the endotracheal tube (ETT) with a tracheostomy tube (TT). 30 years later, little has changed. The question must be asked—are we asking the correct questions? We of course concur that mortality, duration of ventilation, and LOS are important outcomes when determining timing of tracheostomy. Respectfully, we feel that perhaps the patient’s perspectives have been lost in this growing mountain of early versus late tracheostomy studies. Perhaps it is time to consider other outcomes that may be just as important to the patient.

Recent smaller single-centre studies with active allied health input for communication, swallowing and mobility in the ICU have demonstrated significant benefits of an earlier tracheostomy in patient-centred outcomes such as being able to talk, return to eating and drinking, and out-of-bed mobility [3, 4]. Larger data analysis is not possible at this point as such data have not yet been widely collected. And this dearth of data needs to be addressed.

Looking at anatomy and physiology in a little more detail, we know that an ETT passes via the upper airway and renders it obsolete for communication and swallow purposes. An ETT is known to cause damage to the upper airway [5] with 83% prevalence of laryngeal injury found by Brodsky and colleagues [6]. The severity of laryngeal injury was found to increase significantly with an increasing duration of cannulation. The prevalence and severity could also be dependent on the size and material of the ETT, and perhaps also patient mobility whilst cannulated. These potential laryngeal consequences remain once ETT is replaced with a tracheostomy. However, once TT is placed, the upper airway is free from tubing and its capacity is returned. It could be comparable to a plaster cast coming off a limb—we don’t just leave it ‘sitting’, we start mobilising it. The same principle should apply to the upper airway that has been stented open with an ETT, and probably damaged in the process. It should be assessed and rehabilitated. For adequate motor response, sensory stimulation is needed. Airflow is an essential part of sensory information in the upper airway. Without that airflow it is difficult for the patient to recognise the presence and amount of saliva, often impacting swallowing. Therefore, a TT where the cuff stays inflated and no other methods are used to restore some airflow via the upper airway is causing further desensitisation and deconditioning of the upper airway. This is especially important in patients with a neurological injury (as in the cohort in Premraj et al. study), where the disease itself often causes communication and swallowing difficulties, in addition to potential insult from the presence of ETT or TT itself.

It is therefore apparent that for the patient’s upper airway to benefit from an early tracheostomy rehabilitation must commence as soon as possible [7]. The optimal and most natural option is by using a one-way valve [7–9]. If that is not feasible for some reason, then enabling some airflow by using above cuff vocalisation [7, 10, 11] is the next best option. Third, although with its limitations, is leak speech [7]. All these methods, when used after a thorough upper airway patency assessment [12], have shown to safely restore the airflow via the patient’s upper airway, enabling the use of voice and facilitating oral intake. All that even whilst the patient is still receiving support from the ventilator. None of these interventions are possible with an ETT in situ.

We are certainly not claiming superiority of early tracheostomy but are suggesting the focus of the debate to move beyond the age-old outcomes of mortality and length of stay. Patients want to thrive, not just survive. Patients want to speak and to eat. This is universal. Patient-centred outcomes should be collated and analysed to determine if restoring patient’s upper airway physiology faster may result in improved outcomes. These outcomes can only show a difference though, when patients’ upper airway is assessed, and rehabilitation commences as soon as possible after the insertion of a tracheostomy. This is generally a role for the Speech Pathologist—an established position in some ICUs, but certainly still evolving in most. Without specialist input, often the advantages of tracheostomy are not being utilised. In which case, perhaps there are no benefits to having one’s upper airway free from tubing.

## Data Availability

Not applicable.
